# Predicting Novel Features of Toll-Like Receptor 3 Signaling in Macrophages

**DOI:** 10.1371/journal.pone.0004661

**Published:** 2009-03-02

**Authors:** Mohamed Helmy, Jin Gohda, Jun-ichiro Inoue, Masaru Tomita, Masa Tsuchiya, Kumar Selvarajoo

**Affiliations:** 1 Institute for Advanced Biosciences, Keio University, Tsuruoka, Japan; 2 Systems Biology Program, School of Media and Governance, Keio University, Fujisawa, Japan; 3 Division of Cellular and Molecular Biology, Institute of Medical Science, University of Tokyo, Tokyo, Japan; New York University School of Medicine, United States of America

## Abstract

The Toll-like receptor (TLR) 3 plays a critical role in mammalian innate immune response against viral attacks by recognizing double-stranded RNA (dsRNA) or its synthetic analog polyinosinic-polycytidylic acid (poly (I∶C)). This leads to the activation of MAP kinases and NF-κB which results in the induction of type I interferons and proinflammatory cytokines to combat the viral infection. To understand the complex interplay of the various intracellular signaling molecules in the regulation of NF-κB and MAP kinases, we developed a computational TLR3 model based upon perturbation-response approach. We curated literature and databases to determine the TLR3 signaling topology specifically for murine macrophages. For initial model creation, we used wildtype temporal activation profiles of MAP kinases and NF-κB and, for model testing, used TRAF6 KO and TRADD KO data. From dynamic simulations we predict i) the existence of missing intermediary steps between extracellular poly (I∶C) stimulation and intracellular TLR3 binding, and ii) the presence of a novel pathway which is essential for JNK and p38, but not NF-κB, activation. Our work shows activation dynamics of signaling molecules can be used in conjunction with perturbation-response models to decipher novel signaling features of complicated immune pathways.

## Introduction

Toll-Like Receptors (TLRs) play a major role in innate immunity, the first line of mammalian defense against invading pathogens and crucial for antigen-specific acquired immunity development [1], [2]. Upon the recognition of pathogen-associated molecular patterns (PAMPs), such as bacterial lipopolysaccharide (LPS) and viral dsRNA, the 13 currently known TLRs trigger predominantly the activation of MAP kinases and several key transcription factors including nuclear factor-κB (NF-κB), activator protein (AP)-1 and interferon regulatory factor (IRF)- 3 and 7. This results in the induction of numerous proinflammatory cytokines and type I interferons [3], [4], [5]. The dysregulation of TLR signaling, therefore, has been attributed to the pathogenesis of major pro-inflammatory illnesses such as the autoimmune diseases [6], [7].

TLR3, one of the 4 known intracellular members of TLR family, recognizes dsRNA and poly (I∶C) [3], [5], [8] triggers an innate response independent of the adaptor protein Myeloid Differentiation factor 88 (MyD88), which is required for all other TLRs [8], [9]. The specificity of TLR3 response is possibly due to the occurrence of an alanine residue in a critical region of its cytoplasmic domain unlike the proline residue utilized by MyD88 found in other TLRs [5]. Thus, TLR3 initiates its response depending only on the adaptor protein TIR domain-containing adapter-including interferon-β (TRIF) [2], [10]. The recruitment of TRIF mediates the signaling process through the activation of key transcription factors NF-κB, AP-1, IRF-3 and 7 [3], [5], [8]. Although the signaling molecules and their cascades have been broadly investigated, the dynamic outcome of signal transduction between wildtype and genetic mutations still remains poorly understood.

Here, we began the investigation of TLR3 pathway by literature/database curation of the signaling topology in murine macrophages. Next, we analyzed the temporal experimental data of wildtype, TNF Receptor Associated Factor (TRAF)-6 and NFRSF1A-associated via death domain (TRADD)-deficient murine macrophages with poly (I∶C) stimulation [11]–[12] by developing a computational TLR3 model based on perturbation-response approach. This approach does not require the detailed reaction kinetics of each reaction in the signaling topology (as in bottom-up approaches), but rather, considers the activation of signaling molecules as linear response events [13]. Similar modeling approaches have been previously used to infer important biological network features; inferring feedback control of IKK activity in tumour-necrosis factor (TNF) stimulation [14], uncovering switching behaviour of MAPK signaling between epidermal growth factor (EGF) and neuronal growth factor (NGF) stimuli [15], detecting connectivities of reaction molecules [16], predicting missing molecules in TLR4 signaling [17] and *signaling flux redistribution* (*SFR*) at pathway junctions [18].

Our TLR3 model simulations were compared with temporal experimental data of NF-κB, JNK and p38 in three experimental conditions; wildtype, TRAF6-deficient and TRADD-deficient macrophages. Collectively, the results suggest i) the existence of novel intermediary steps (e.g. missing cellular processes, proteins or phosphorylation states) between extracellular poly (I∶C) stimulation and intracellular TLR3 binding, and ii) the presence of a novel pathway which is essential for JNK and p38 activation.

## Results and Discussion

### Determination of the TLR3 signaling topology in macrophages

TLR3 is expressed in several cell types including macrophages, murine embryonic fibroblasts (MEFs) and dendritic cells (DCs), however, it was not found in B Cells, T Cells and NK cells [8], [19], [20], [21], [22]. Moreover, the experimental observations of TLR3 signaling under various genetic knock-outs show controversial roles of certain molecules in different cell types. For instance, Gohda *et al.* [11] showed that TRAF6 is dispensable for TLR3 induced NF-κB in poly (I∶C) stimulated macrophages, while Jiang *et al.* [23] showed the requirement of TRAF6 to NF-κB activation in MEFs. Also, for NF-κB and MAP kinases activation, TRADD is not critical in macrophages, whereas, it is important for MEFs [12], [24]. These data indicate that there is no common topology for TLR3 signaling and, therefore, we cannot combine data obtained from different cell types to create unified TLR3 signaling topology. Instead, independent cell type analysis should be performed.

**Figure 1:**
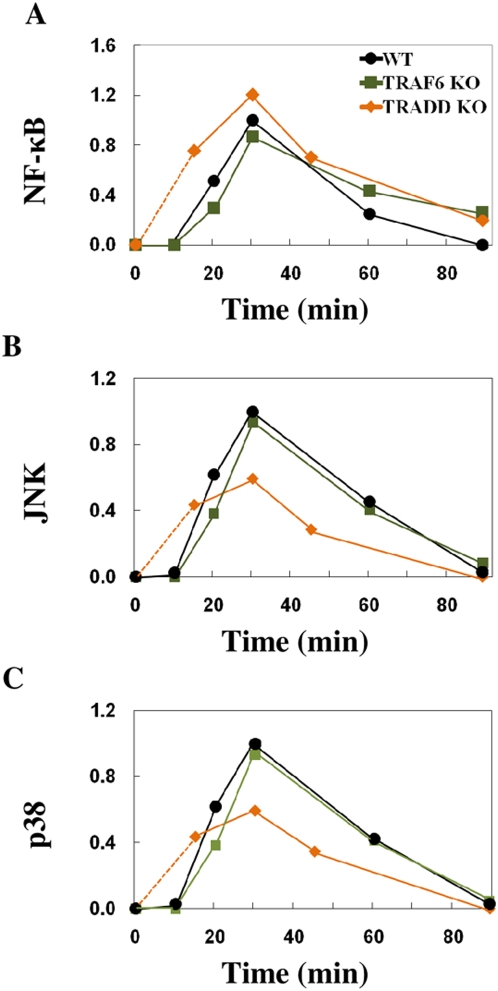
NF-κB and JNK experimental activation profiles. A), B) and C) show experimental activation profiles of NF-κB, JNK and p38, respectively in (WT) (black), TRAF6 KO (green), TRADD KO (orange) obtained from [11], [12]. The activation levels were quantified from the western blots using ImageJ [http://rsbweb.nih.gov/ij/]. The *x*-axis represents the time in minutes and the *y*-axis represents the relative activation profile. Note: As TRADD KO data is unavailable at 10 min (earliest at 15 min), we could not observe delayed activation of as noted for WT and TRAF6 KO. Therefore, we used dotted line to connect 0 min and 15 min time points.

Here, we investigate the signaling topology for murine macrophages only. It is known that TRIF interacts with TLR3 at the TIR domain and TRAF6 binds to the N-terminal of TRIF [8], [11], [25]. However, we found, in murine macrophages, the role of TRAF6 in TLR3 signaling is dispensable [11]; the temporal experimental profiles of MAP kinases (JNK and p38) and NF-κB activation to poly (I∶C) stimulation in TRAF6 KO were only slightly reduced compared with wildtype levels (Figure 1A–C WT and TRAF6 KO).

**Figure 2:**
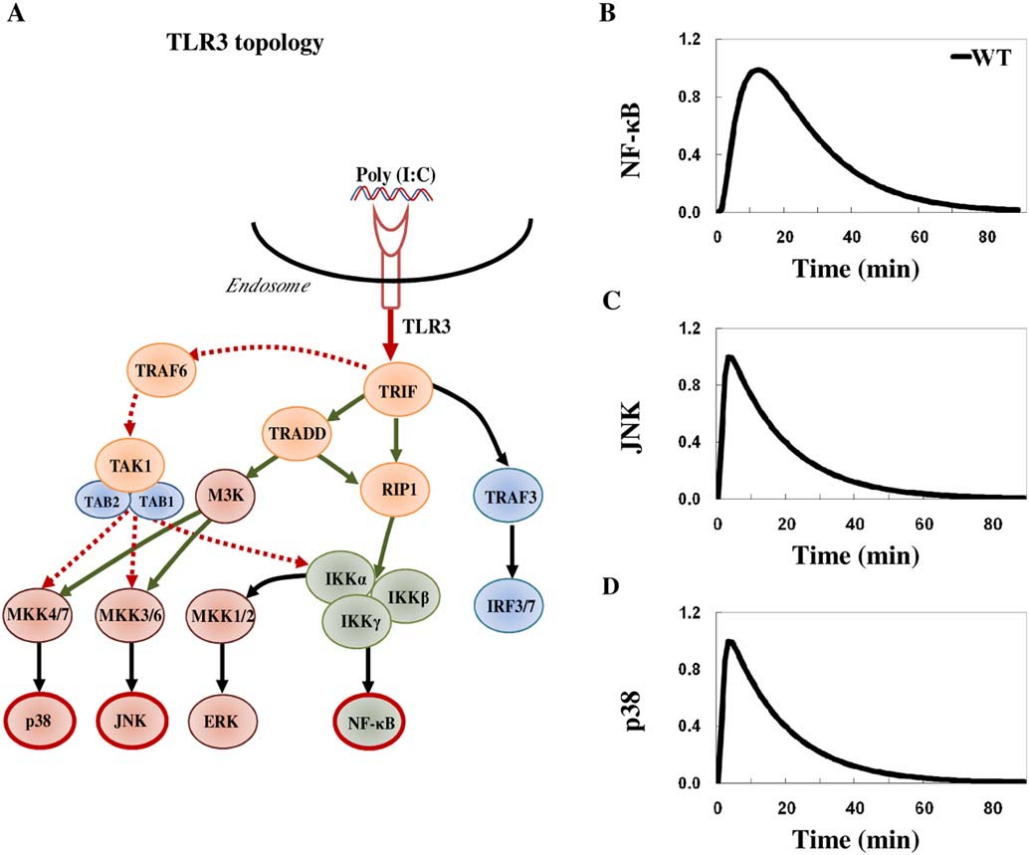
Analysis of TLR3 pathway in macrophages. A) Schematic representation of the determined TLR3 pathway topology in macrophages. dsRNA or poly (I∶C) stimulated TLR3 triggers TRIF dependant response by the recruitment of TRIF to the cytoplasmic domain of the receptor which then allows RIP1, TRAF6, TBK1 and TRAF3 to bind with TRIF. This results in the activation of MAP kinases (MKK1/2, MKK3/6 and MKK4/7) and IκB kinase complex; MKK1/2, MKK3/6 and MKK4/7 activate ERK, JNK and p38, respectively and IκBα degradation releases NF-κB. TBK1 phosphorylates IRF-3 and 7. ERK, JNK and p38 translocate to the nucleus and activate the transcription factor AP-1, and NF-κB, IRF-3 and IRF-7 translocate to the nucleus. AP-1 and NF-κB bind to the promoter regions of cytokine genes such as *Tnf* and *Il6* while IRF-3, IRF-7 together with NF-κB bind to the promoter region of chemokine genes such as *Cxcl10* and *Ccl5* and induce their transcription. Protein-protein interactions between molecules at the two signaling branches analyzed are highlighted in brown and blue. The dotted lines indicate weak activation (see maintext). B), C) and D) show simulations of NF-κB, JNK and p38 activation, respectively, in wildtype (WT). The *x*-axis represents the time in minutes and the *y*-axis represents the relative activation profile.

Recently, TRADD has been shown to be involved in the TLR3 signaling [11], [24], [26]. TRADD-deficient macrophages showed downregulation of MAP Kinases and a slight upregulation of NF-κB activation (Figure 1A–C TRADD KO) [11]. RIP1, which binds to TRIF at C-terminal (RHIM domain), interacted with TRADD through death domain (DD) suggesting TRADD's possible involvement in RIP1 ubiquitination [12]. Since RIP1 is required for TRIF-dependent signaling in response to poly (I∶C) stimulation [27], TRADD is likely involved in NF-κB activation via RIP1. Putting together, we created a macrophage TLR3 signaling topology (Figure 2A).

### The prediction of missing intermediary cellular processes in poly (I∶C) stimulated macrophages

So far, we have built the signaling topology for TLR3 pathways in macrophages. Next, to understand the complex dynamic interplay of the various intracellular signaling molecules in the regulation of NF-κB, JNK and p38 in poly (I∶C) stimulation, we developed a computational model of the TLR3 signaling (see Materials and Methods). Each signaling response (i.e., the rate of activation of each signaling molecule) in the model is represented by 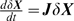, where δ***X*** is relative activated concentration of signaling molecules and the parameters (elements of ***J***) are chosen to fit the semi-quantitative experimental profiles (e.g, western blots, EMSA, etc.) of NF-κB, JNK and p38 of wildtype macrophages stimulated with poly (I∶C) (Figure 1A–C WT). As mentioned earlier, TRAF6 KO does not noticeably affect NF-κB, JNK or p38 activation compared to wildtype (Figure 1A–C). Hence, in the model, we used a low parameter value between TRIF and TRAF6 to limit the response flux propagation through TRAF6 such that the removal of TRAF6 in the model will only slightly affect its downstream reactions (see Materials and Methods for details).

We, next, simulated activation of NF-κB, JNK and p38 in wildtype. Our results show the time to reach peak values were 15–20 min earlier than in actual experiments (Figure 2B–D). Clearly, this model could not explain the delayed experimental activation of NF-κB, JNK and p38. We can consider time delay processes of signaling events are due to missing molecules/complex formation or spatial movement of molecules [13]. The deterministic kinetic evolution equation used in our model, non-linear ***F*** in general (Eq.1, Materials and Methods), can include such information by setting total derivative of time to partial derivative in time and space. In other words, Jacobian matrix ***J*** can contain temporal and spatial information of the network process. Since we are not performing spatial simulation, time delay response can be lumped as missing molecules/processes in the network. For example, using a TLR4 model, we previously predicted the delayed activation of TRIF-dependent pathways by using a number of additional response reactions representing missing signaling features (molecules/processes, spatial movements or complex formation) in the original network [17]. Our result was later substantiated by McGettick *et al.*, 2006 [28] who demonstrated two novel signaling molecules (PKCε and TRAM) act upstream of TRIF and, recently, by Kagan *et al.*, 2008 [29] who discovered that the internalization of TLR4 into the endosome is required prior to TRIF-dependent pathway activation. Thus, the delayed activation of NF-κB and MAP Kinases in poly (I∶C) stimulation could also be due to missing molecules or cellular processes.

**Figure 3:**
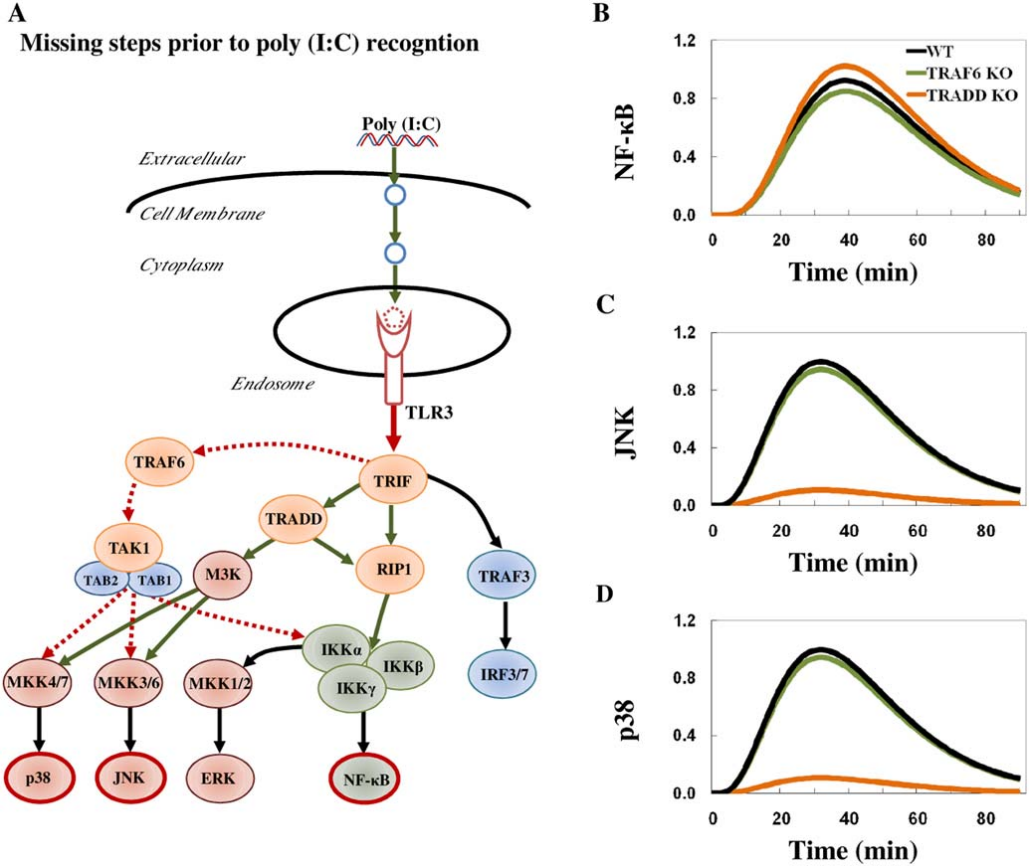
Prediction of missing steps prior to poly (I∶C)/TLR3 binding. A) Schematic representation of TLR3 model after adding three signaling intermediates upstream of TLR3 representing uncharacterized cellular processes (blue) and TLR3-ectodomain dimerization (red dotted) (see maintext). Note: from our model, it is not possible to equate the three intermediary steps to represent exactly three actual biological events, since spatial transport processes might be one of candidates for the time delay. B), C) and D) show simulations of NF-κB, JNK and p38 activation, respectively, in the wildtype (WT) (black), TRAF6 KO (green), TRADD KO (orange). The *x*-axis represents the time in minutes and the *y*-axis represents the relative activation profile.

To investigate and locate the likely position of the necessary missing intermediary steps in the TLR3 pathway predicted by the model, we further surveyed the literature. TRIF directly bind to the TIR domain of TLR3 and does not require TRAM for its activation [30]. Thus, it is unlikely that missing intermediary steps exist between TLR3 and TRIF. Furthermore, TLR3 KO and TRIF KO both showed similar response; abolished activation of NF-κB and MAP Kinases [30]. These data led us to hypothesize the missing intermediary steps (e.g. signaling molecules or processes) are upstream of TLR3 and we updated our model to begin simulation not from TLR3, but rather from poly (I∶C) downwards (Figure 3A). We also represented each new uncharacterized molecule/process as a signaling intermediate in our model.

To obtain the delayed activation of NF-κB and MAP kinases in accordance with experimental data of wildtype, that is, null activation till 10 min, peak values around 30 min and reduced activation after 60 min for all molecules (Figure 1A–C), we were required to add three intermediary steps (signaling intermediates) (Figure 3B–D WT). Having less or more intermediary steps resulted in peak value reaching faster or slower than experimental peak, respectively (data not shown).

While preparing this manuscript, Liu *et al.* demonstrated that TLR3-ectodomains dimerizes before signal propagation [31]. Our prediction of novel intermediary steps in upstream of TLR3 activation indicates that TLR3-ectodomains dimerization could be one of the missing intermediary steps. The other intermediary steps could indicate the endocytosis of poly (I∶C) and its subsequent transport mechanisms to be recognized by TLR3.

### The existence of missing pathway for MAP Kinases activation in poly (I∶C) stimulation

To further test the predictive capability of our model, we simulated TRAF6 KO and TRADD KO (see Materials and Methods). We wondered whether the same TLR3 model could successfully simulate the activation of NF-κB, JNK and p38 in all three conditions: wildtype, TRAF6 KO and TRADD KO. To create KO condition from wildtype model, we set the reactions of the KO molecule null, while all other model parameters retain their original wildtype values.

The TRAF6 KO simulations matched the experimental data, i.e., the removal of TRAF6 only slightly downregulated NF-κB, JNK and p38 activation (Figure 3B–D TRAF6 KO). For TRADD KO, NF-κB activation was slightly increased in experiments (Figure 1A). This result was recapitulated by our model which suggests the enhancement of NF-κB activation in TRADD KO is due to *signaling flux redistribution* [18] (Figure 3B TRADD KO). However, in contrasts to experimental results, which showed only a small downregulation, simulations of JNK and p38 showed almost abolished activation (Figure 3C–D TRADD KO). Thus, our simulation failure suggests, in actual cells, a novel pathway might exist that compensate the loss of MAP Kinases activation in TRADD KO.

**Figure 4:**
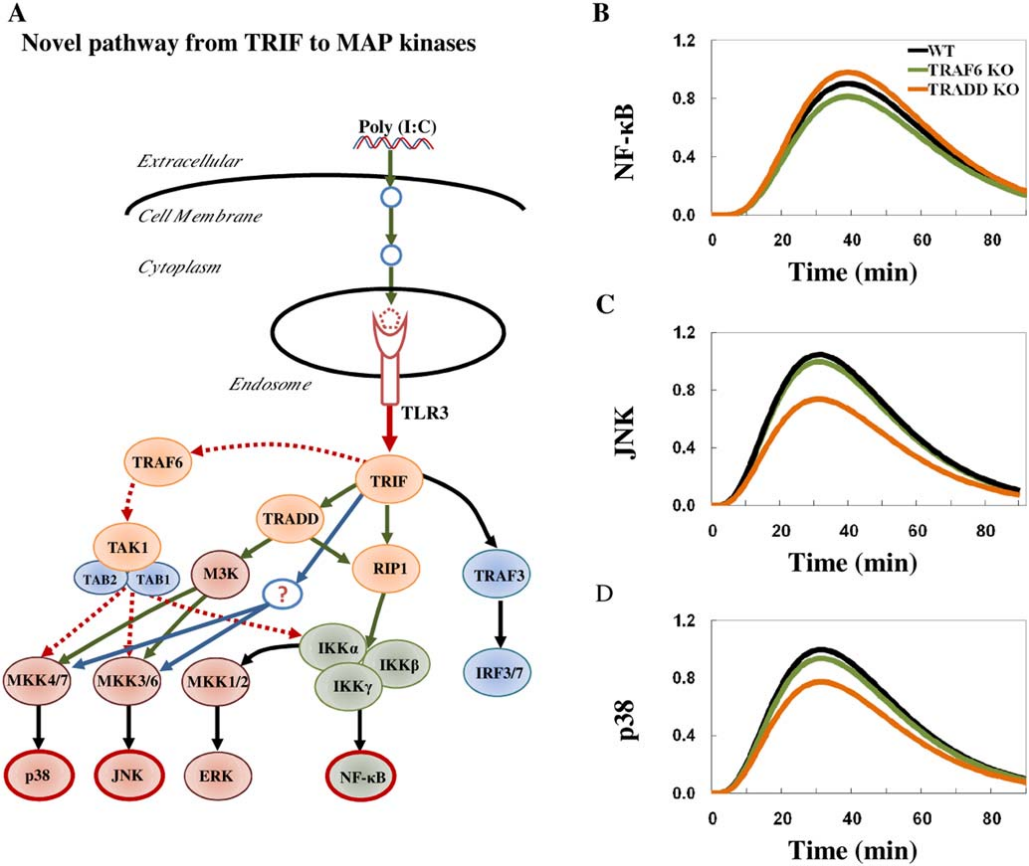
A novel pathway is crucial for MAP kinases activation in poly (I∶C) stimulated macrophages. A) Schematic representation of the final TLR3 model after adding novel pathway (blue) from TRIF activates MAP kinases. B), C) and D) show simulations of NF-κB, JNK and p38 activation, respectively, of the final macrophages TLR3 model with novel pathway, in wildtype (WT) (black), TRAF6 KO (green) and TRADD KO (orange). The *x*-axis represents the time in minutes and the *y*-axis represents the relative activation profile.

Macrophages with TRIF mutation treated with poly (I∶C) showed abolishment of MAP kinase ERK activation [32] and all MAP kinases showed similar kinetics in wildtype and TRAF6 KO [11]. Thus, we added a pathway from TRIF to MAP kinases into our model (Figure 4A and Table 1) and adjusted the parameter values between TRIF to RIP1, TRADD and the novel pathway to fit wildtype NF-κB, JNK and p38 activation. We re-performed the simulations of TRAF6 KO and TRADD KO. The final model simulations matched experimental outcome for all three conditions (Figure 4B–D).

**Table 1:** The final *in silico* TLR3 model reactions and parameter values.

No	Reaction	Formula	Parameter value (1/s)	Remarks
**1**	IM1→IM2	k1*IM1	k1 = 0.002	Novel intermediates acting upstream of TLR3 representing uncharacterized cellular events.
**2**	IM2→IM3	k2*IM2	k2 = 0.002	
**3**	IM3→TLR3	k3*IM3	k3 = 0.002	
**4**	TLR3→TRIF	k4*TLR3	k4 = 0.001	TLR3 recruits TRIF.
**5**	TRIF→TRAF6	k5* TRIF	k5 = 0.002	TRAF6 binds to TRIF.
**6**	TRIF→IM4	k6*TRIF	k6 = 0.004	TRIF activates JNK through Novel pathway
**7**	TRIF→RIP1	k7*TRIF	k7 = 0.007	RIP1 binds to TRIF.
**8**	TRIF→TRADD	k8*TRIF	k8 = 0.002	TRADD binds to TRIF.
**9**	TRIF→TRAF3	k9* TRIF	k9 = 0. 01	TRAF3 binds to TRIF.
**10**	TRAF6→TAB/TAK	k10*TARF6	k10 = 0.04	TRAF6 binds to TAB/TAK complex.
**11**	RIP1→IKK	k11*RIP1	k11 = 0.04	RIP1 activated IKK complex.
**12**	TRADD→RIP1	k12*TRADD	k12 = 0.0001	RIP1 ubiquitination through TRADD.
**13**	TRADD→MKKK	k13*TRADD	k13 = 0.04	TRADD activates JNK and p38 corresponding MKKK.
**14**	MKKK→MKK3/6	k14*MKKK	k14 = 0.04	MKKK activates JNK through activation of MKK3/6.
**15**	MKKK→MKK4/7	k15*MKKK	k15 = 0.04	MKKK activates p38 through activation of MKK4/7.
**16**	TAB/TAK→MKK4/7	k16* TAB/TAK	k16 = 0.03	Activation of MAP kinases and IKK via TAB/TAK complex.
**17**	TAB/TAK→MKK3/6	k17*TAB/TAK	k17 = 0.007	
**18**	TAB/TAK→IKK	k18* TAB/TAK	k18 = 0.9	
**19**	IM4→MKK3/6	k19* IM4	k19 = 0.04	The novel pathway activates JNK.
**20**	IM4→MKK4/7	k20* IM4	k20 = 0.04	The novel pathway activates p38.
**21**	IKK→NF-κB/IκB	k21*IKK	k21 = 0.00167	Phosphorylation of IκBα/NF-κB via IKK complex.
**22**	IKK→p105/Tp12	k22*IKK	k22 = 0.0009	Activation of ERK via IKK.
**23**	p105/Tp12→MKK1/2	k23* p105/Tp12	k23 = 0.003	
**24**	NF-κB/IκB→NF-κBc	k24* NF-κB/IκB	k24 = 0.0333	Release of NF-κB after IκB phosphorylation.
**25**	NF-κBc→NF-κBn	k25* NF-κBc	k25 = 1.0	NF-κB translocate to the nucleus.
**26**	NF-κBn→NF-κBdeg	k26* NF-κBn	k26 = 0.99	NF-κB degradation.
**27**	MKK1/2→ERKc	k27*MKK1/2	k27 = 0.0167	Activation of ERK via MKK1/2.
**28**	ERKc→ERKn	k28* ERKc	k28 = 0.99	ERK translocate to the nucleus.
**29**	ERKn→AP-1	k29* ERKn	k29 = 0.99	ERK activates AP1.
**30**	MKK3/6→JNKc	k30*MKK3/6	k30 = 0.4	Activation of JNK via MKK3/6.
**31**	JNKc→JNKn	k31*JNKc	k31 = 1.0	JNK translocate to the nucleus.
**32**	JNKn→AP-1	k32* JNKn	k32 = 0.99	JNK activates AP1.
**33**	MKK4/7↔p38c	k33*MKK4/7	k33 = 0.4	Activation of p38 via MKK4/7.
**34**	p38c→p38n	k34* p38c	k34 = 1.0	p38 translocates to the nucleus.
**35**	p38n→AP-1	k35* p38n	k35 = 0.99	p38 activates AP1.
**36**	AP1→AP-1deg	k36* AP1	k36 = 0.085	AP1 degradation.
**37**	TRAF3→TBK1	k37*TRAF3	k37 = 0.0001	TRAF3-TBK1 interaction.
**38**	TBK1→IRF-3/7c	k38*TBK1	k38 = 0.333	IRF3/7 binds TBK1.
**39**	IRF-3/7c→IRF-3/7n	k39*IRF-3/7c	k39 = 0.000167	IRF3/7 translocate to the nucleus.
**40**	IRF-3/7n→IRF-3/7deg	k40*IRF-3/7n	k40 = 0.0001	IRF3/7 degradation.

To investigate other possible pathways for JNK and p38 activation in poly (I∶C) stimulation, reports indicate two other receptors known to recognize dsRNA; the retinoic-acid-inducible protein (RIG)-I and melanoma-differentiation-associated gene (MDA) 5 and, therefore, might be potential candidates [33], [34], [35]. However, firstly, RIG-I is unable to recognize poly (I∶C) [33], [35] and secondly, MDA5 signaling pathway is unknown to trigger MAP kinases [35]. Furthermore, TRIF mutation showed abolishment of ERK activation [32] and all MAP kinases seem to possess similar kinetics in poly (I∶C) stimulation [11]. Taken together, these data suggest the predicted pathway for JNK and p38 activation is through TRIF and not by RIG-1 or MDA5.

To find the possible candidates to be involved in the novel pathway, we again surveyed the literature. The TRAF family members, six to-date, are well-known to bind to the TIR domain of TRIF with their C-terminal [36], [37], [38], [39], while two members of the RIP family, RIP1 and RIP3, are found to interact with TRIF through the RHIM domain found in RIP1, RIP3 and TRIF [40], [41]. RIP1, TRAF6 and TRAF3 already exist in the current TLR3 signaling, while TRAF1 was recently found to inhibit the TRIF-mediated signaling [38], [39]. Thus, we suggest that RIP3, TRAF2, TRAF4 or TRAF5 may be part of the novel pathway activating JNK and p38.

In summary, the analysis of the poly (I∶C) stimulation in macrophages reveals the involvement of uncharacterized missing intermediary steps prior to TLR3 activation and the existence of a key pathway from TRIF to JNK and p38, but not NF-κB activation. Although further experimental validation is required to identify the uncharacterized processes and molecules nevertheless, we show that the simple mass-action linear response can represent activation dynamics of the TLR3 signaling, and can be used to predict novel features of complicated immune pathways.

## Materials and Methods

### Development of *in silico* TLR3 model using perturbation-response approach

The basic principle behind our perturbation-response approach is to induce a controlled perturbation of a certain (input) reaction molecule of a system, which is kept at steady-state, and monitor the response of the concentration/activation levels of other molecules (output) of the system. By finding a relationship between the input perturbation (e.g. poly (I∶C) stimulation) and output response (e.g. JNK, NF-κB activation), the mechanistic properties of the system such as network topology can be uncovered (e.g. novel TRIF-MAP Kinases pathway) without knowing each individual's detailed kinetics.

To illustrate our approach, let us perturb a stable biological network consisting of *n* molecules from reference (stable) steady-state. The deterministic kinetic evolution equation is
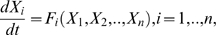
(1)where the corresponding vector form of Eq. 1 is 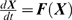. ***F*** is a vector of any non-linear function of the molecules vectors ***X*** = (*X_1_*, *X_2_*, ‥, *X_n_*), which represents activated concentration levels of signaling molecules (for example through phosphorylation, binding concentration of transcription factors to promoter regions etc.). The response to perturbation can be written by ***X*** = ***X***_0_+δ***X***, where ***X***_0_ is reference steady-state vector and δ***X*** is relative response from the steady-states (δ***X*** *_t_*_ = *0*_ = **0**).

The use of small perturbation around steady-state leads to important simplification to the evolution equation, which can be highly non-linear (Eq. 1), resulting in the approximation of the first-order term. In vector form, 
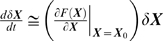
, noting that zeroth order term ***F***(***X***_0_) = 0 at the steady-state ***X***_0_ and the *Jacobian* matrix or linear stability matrix, 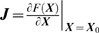. The elements of *J* are chosen by fitting δ***X*** with corresponding experimental profiles and knowing the network topology (e.g., activation causality). Hence, the amount of response (flux propagated) along a signaling pathway can be determined using the law of mass flow conservation with *first order mass-action kinetics*, 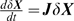 (Table 1) [13], [18], [42]. We considered all signaling response to be linear and sequential up to 90 min after poly (I∶C) stimulation (equation [2]) as apparent from the formation-depletion activation profiles (see Experiments, and Results and Discussion). Although there will not be any issue for parameter sensitivity in our system because of linear events, multiple solutions of parameter space, ***J***s, can occur [13]. To overcome this, we compare our (wildtype) model with two other (TRAF6 KO and TRADD KO) experimental conditions (see Verification of Model Parameters).

### Modeling strategy

**Figure 5:**
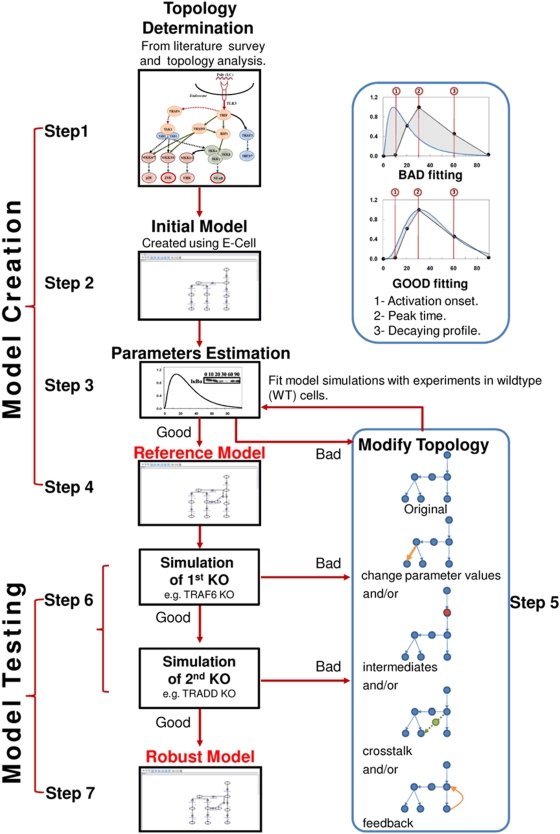
The modeling strategy. See maintext for details.

We first curated the murine macrophage TLR3 signaling topology from current literature/database sources and by checking with relevant experimental data (Figure 5, step 1 and see Results and Discussion) [11], [12], [31], [24], [40], [41], [43]. Next, using the topology, we developed a dynamic model of TLR3 signaling on the E-Cell simulation platform [44] to simulate the dynamics of NF-κB and JNK activation (Figure 5, step 2). The parameters of our model (elements of Jacobian matrix ***J***) were estimated by fitting the simulation profiles of NF-κB, JNK and p38 with corresponding activation profiles semi-quantified from immunoblots, obtained from wildtype macrophages [11] (Figure 5, step 3) (Note that our model works quantitatively if appropriate data is available). If the model successfully fits the wildtype profile of NF-κB and JNK, we accept the model and call it the Reference Model (Figure 5, step 4). Otherwise, if the model fails, we adjust the parameters values or model topology until the model is able to simultaneously fit the experimental profiles of NF-κB, JNK and p38 reasonably (Figure 5, step 5).

The model simulation results were deemed acceptable by comparing the simulation profile with the experimentally semi-quantitative profile based on three criteria; i) time of activation onset, ii) time to reach peak, and iii) decay profile (Figure 5 insert). For example, JNK experimental profile shows null activation until 10 min, peak activation at 30 min and decaying trend until 90 min (Figure 1B, WT). In our first simulation of JNK (Figure 2C, WT), the time of activation onset was almost instantaneous, peak at around 10 min, and complete decay after 60 min. This simulation, therefore, is considered BAD based on the three criteria which poorly matched (Figure 5 insert, upper panel). On the other hand, after modifying the signaling topology by adding novel intermediary steps, the simulation improved on all three criteria and considered GOOD (Figure 3C, WT, and Figure 5 insert, lower panel).

### Verification of Model Parameters

There can be several parameter spaces for Jacobian matrix, ***J***s, which could fit wildtype semi-quantitative activation profiles of NF-κB, JNK and p38. To reduce the parameter spaces, we performed an iterative two-mode process of model “creation” and “testing”; “Creation” is to develop and fit our model with published available semi-quantitative profiles of NF-κB and MAP kinases activity in wildtype (step 4). “Testing” is to optimize reaction networks and parameter values, obtained from step 4, by simulating the knockout (KO) conditions (TRAF6 KO and TRADD KO) and comparing the results with their respective experimental profiles of NF-κB and MAP kinases activation [11], [12]. Each KO model was generated from the wildtype model by setting the parameters involving the KO molecule null. If our KO simulation does not compare well with experiments, we re-tune the model parameters till the model simulations fit the activation profiles of both wildtype and TRAF6 KO (Figure 5 step 6). If unsuccessful, we modify the topology so that a better fit can be obtained, for example, adding novel signaling intermediates to obtain the delayed activation [17] or crosstalk mechanism to provide an alternative source of activation in the KO condition [42], [45], [46] (Figure 5 step 4, see Results and Discussion). To avoid false prediction, we further check comparing the model simulation against TRADD KO. When the model fails at these steps, it implies that either ***J*** (parameter space) is not correct or the represented topology is insufficient. We repeat the procedure of modifying parameter values and topology until we obtained reasonable predictions for all 3 conditions (wildtype, TRAF6 KO and TRADD KO) at 6 time points (0, 10, 20, 30, 60 and 90 min). At this point, we accepted the model and called it the Robust Model (Figure 5 step 7). Therefore, using steps 1–7, we were able to identify missing or incorrect feature(s) of the TLR3 pathways. The final (robust) TLR3 model's equations and parameter values can be found in Table 1.

### Final TLR3 Response Model

Our final model consists of 34 molecules with 40 reactions and parameters developed after comparing with dynamics of 3 molecules (NF-κB, JNK and p38) at 6 time points (0, 10, 20, 30, 60 and 90 min) for 3 conditions (wildtype, TRAF6 KO and TRADD KO) (Table 1).

### Experiments on macrophages

We utilized our published time-course experimental data of NF-κB, JNK and p38 of wildtype (model creation) and TRAF6-deficient (model testing) macrophages with 10 µg/ml poly (I∶C) stimulation [11]. For further model testing, we utilized published experimental profile of NF-κB, JNK and p38 of TRADD-deficient macrophages with similar poly (I∶C) stimulation [12]. The activation levels of NF-κB, JNK and p38 (Figure 1A–B, Figure S1A) were quantified from the western blots using ImageJ (http://rsbweb.nih.gov/ij/).
